# Quantitative Tandem Affinity Purification, an Effective Tool to Investigate Protein Complex Composition in Plant Hormone Signaling: Strigolactones in the Spotlight

**DOI:** 10.3389/fpls.2018.00528

**Published:** 2018-04-26

**Authors:** Sylwia Struk, Lukas Braem, Alan Walton, Annick De Keyser, François-Didier Boyer, Geert Persiau, Geert De Jaeger, Kris Gevaert, Sofie Goormachtig

**Affiliations:** ^1^Department of Plant Biotechnology and Bioinformatics, Ghent University, Ghent, Belgium; ^2^Center for Plant Systems Biology, VIB, Ghent, Belgium; ^3^Department of Biochemistry, Ghent University, Ghent, Belgium; ^4^Center for Medical Biotechnology, VIB, Ghent, Belgium; ^5^UMR 1318, Institut National de la Recherche Agronomique – Institut Jean-Pierre Bourgin, Versailles, France; ^6^Institut de Chimie des Substances Naturelles – UPR 2301, Centre de Recherche de Gif, Centre National de la Recherche Scientifique, Paris, France

**Keywords:** strigolactones, SMXL7, qTAP, plant hormone signaling, protein dynamics

## Abstract

Phytohormones tightly regulate plant growth by integrating changing environmental and developmental cues. Although the key players have been identified in many plant hormonal pathways, the molecular mechanisms and mode of action of perception and signaling remain incompletely resolved. Characterization of protein partners of known signaling components provides insight into the formed protein complexes, but, unless quantification is involved, does not deliver much, if any, information about the dynamics of the induced or disrupted protein complexes. Therefore, in proteomics research, the discovery of what actually triggers, regulates or interrupts the composition of protein complexes is gaining importance. Here, tandem affinity purification coupled to mass spectrometry (TAP-MS) is combined with label-free quantification (LFQ) to a highly valuable tool to detect physiologically relevant, dynamic protein–protein interactions in *Arabidopsis thaliana* cell cultures. To demonstrate its potential, we focus on the signaling pathway of one of the most recently discovered phytohormones, strigolactones.

## Introduction

Plants produce a broad range of phytohormones, which are small molecules that regulate their growth and development and control their responses to biotic and abiotic stresses, locally as well as throughout the entire plant. Although phytohormones have been intensively studied, a lot remains to be resolved about the mechanisms underlying their mode of action (Černý et al., 2016). Proteins that are crucial for the perception and the transduction of molecular signals, such as phytohormones, often form complexes to fulfill their biological function. Most of the protein–protein interactions (PPIs) are not static, but rather dynamic, because they are constantly subjected to changes in the crowded cellular environment. Knowledge of the interaction partners of a given protein may provide insight into its function at the molecular level or into the process in which it is involved. Although quite some methods exist to detect PPIs, for a better understanding of cellular mechanisms, the identification of functionally relevant PPIs and, in particular, the characterization of how they are influenced by varying physiological conditions are required (Buntru et al., 2016).

Affinity purification techniques coupled to mass spectrometry (AP-MS) are established tools to investigate the spectrum of possible interaction partners of a protein of interest. The proteome-wide insight they offer provides information on both direct as well as indirect interactors. In the plant field, tandem affinity purification (TAP) is probably one of the most successful AP-MS approaches (Dedecker et al., 2015) and has been efficiently used to purify protein complexes from different tissues and from several plant species (Rohila et al., 2006; Van Leene et al., 2007, 2011; Nelissen et al., 2015; Goossens et al., 2016). To execute TAP, a bait protein is fused translationally with a double affinity tag, most commonly the GS-tag, consisting of two immunoglobulin G-binding domains of protein G, combined with a streptavidin-binding peptide, separated by a specific tobacco etch virus (TEV) protease cleavage site (Bürckstümmer et al., 2006; Van Leene et al., 2007) or by a rhinovirus 3C protease cleavage site in the improved version (Van Leene et al., 2015). The protein complex, in which the tagged bait is engaged, is retrieved in two consecutive purification steps under near physiological conditions, whereafter the proteins are digested and identified by means of MS (Van Leene et al., 2015). Two-step purifications generally lead to less complex samples that are relatively free from the unspecific binding proteins in comparison with single-step purifications, thereby allowing a higher resolution view of the members of a complex (Li, 2011). However, TAP-MS typically identifies only stable interactors and is faced with difficulties in the case of proteins interacting with weak affinity (Gavin et al., 2011).

It is becoming increasingly clear that not only knowledge of the interaction partners of a bait protein is important, but also of the conditions under which such interactions occur (Buntru et al., 2016). Proteins involved in plant hormone signaling provide a good example. During auxin signaling, the simple spectrum of all possible interaction partners of the AUXIN/INDOLE-3-ACETIC ACID (AUX/IAA) proteins includes the AUXIN RESPONSE FACTOR (ARF) activators, TOPLESS (TPL), TOPLESS RELATED (TPR) proteins, the generic members of the SCF complex ARABIDOPSIS SKP1-LIKE (ASK1) and CULLIN1 (CUL1), the auxin-related F-BOX protein TRANSPORT INHIBITOR RESPONSE 1 (TIR1), E2 proteins, and ubiquitin. However, this list does not reveal the underlying dynamics, such as the fact that the AUX/IAA proteins interact with the SCF^TIR1^ complex only in the presence of auxin, while otherwise they are linked to ARF activators to repress their transcriptional activity through the action of TPL/TPR proteins (reviewed in Leyser, 2018). Obviously, information on the dynamics of the AUX/IAA complexes and on the effect of auxin is required to gain full insight into the signaling cascade of this hormone. Similar signal-dependent interactions have been identified for jasmonates, brassinosteroids, and ethylene, as well as for other, non-hormone-related dynamic interactions that modulate responses to environmental cues or developmental stages (Larrieu and Vernoux, 2015).

Hence, it is important to study how protein complexes act in response to (different) stimuli. To this end, a technique is required that fulfills three criteria: (i) comprehensive coverage of proteins engaged in a complex; (ii) compatibility with a biological system in which a trigger (stimulus) can be applied; and (iii) a quantitative readout. Therefore, we have developed a quantitative method based on TAP in cell cultures to study protein complex dynamics in *Arabidopsis thaliana*. Label-free quantification (LFQ) is applied to determine shifts in the levels of protein complex members in a trigger-dependent manner, thus mapping the dynamics of the protein complexes. We used the MaxLFQ algorithm integrated into the MaxQuant software (Cox et al., 2014). This comparison of proteome samples avoids stable isotope labeling, of which the metabolic version is somewhat restricted in plants due to very high costs or labeling efficiency issues (Gruhler et al., 2005). LFQ relies on replicates analyses to quantify differences in peptide ion intensities between different samples by means of statistical algorithms. For AP-MS studies, LFQ is based on the observation that most experimental conditions do not influence the abundance of non-specifically interacting proteins, thereby allowing accurate identification of the proteins that interact differentially, for instance, because of a treatment. Thus, in general, LFQ techniques are promising alternatives, because they are cost effective, easy to perform, and suitable for comparative analysis of large numbers of samples (Ramisetty and Washburn, 2011).

We focused on the protein complex involved in strigolactone signaling. This hormone was discovered a decade ago and its essential role in modulating various aspects of above- and below-ground plant architecture has been demonstrated (Smith and Waters, 2012). However, full knowledge of the signaling components is missing. In *Arabidopsis*, the synthetic strigolactone analog *rac-*GR24 is perceived and hydrolyzed by the α/β hydrolase DWARF14 (D14). As a result, D14 undergoes a conformational change (Yao et al., 2016) that allows its interaction with the F-box protein MORE AXILLARY BRANCHES 2 (MAX2), that is part of an SCF E3 ubiquitin ligase complex (Stirnberg et al., 2007; Hamiaux et al., 2012), and with proteins from the SMAX1-like (SMXL) family, which are the most recently described components of the strigolactone pathway (Jiang et al., 2013; Stanga et al., 2013; Zhou et al., 2013). Forward genetics in rice (*Oryza sativa*) revealed that a SMXL homolog, DWARF 53 (D53) is a repressor of the strigolactone signaling involved in tiller number regulation (Jiang et al., 2013; Zhou et al., 2013). Indeed, a gain-of-function *d53* mutant had a high tillering, dwarf phenotype, and was insensitive to the addition of strigolactones (Zhou et al., 2013). The gain-of-function phenotype was caused by the mutation of an amino acid region, resulting in resistance against strigolactone-induced protein degradation (Jiang et al., 2013; Zhou et al., 2013). In *Arabidopsis*, the SMXL family consists of eight members divided into four subclades, from which SMXL6, SMXL7, SMXL8 together with D53 form one phylogenic clade (Stanga et al., 2013). These *Arabidopsis* SMXL proteins were also rapidly degraded by the 26S proteasome upon treatment with *rac*-GR24 in a D14- and MAX2-dependent manner, thereby influencing the shoot and root architecture. Mutation of the amino acid residues of SMXL6 or SMXL7, corresponding to those mutated in the *d53* allele, conferred resistance to *rac*-GR24-dependent degradation. The role of this region and its involvement in the observed resistance to strigolactone-induced degradation remains unclear (Soundappan et al., 2015; Wang et al., 2015). Although the exact molecular function of the SMXL6, SMXL7, and SMXL8 proteins is largely unknown, one role might be related to a conserved ETHYLENE-RESPONSE FACTOR Amphiphilic Repression (EAR) motif (Liang et al., 2016) that allows the interaction with TPL/TPR proteins and subsequently regulates transcription of several genes (Ohta et al., 2001). By contrast, the EAR motif has been suggested to allow interactions between SMXL and other proteins containing a C-Terminal Lissencephaly Homology (CTLH) domain resulting in non-transcriptional responses (Liang et al., 2016).

Here, we aimed at investigating whether the reported changes in the protein complexes formed around SMXL7 could be discovered by means of quantitative TAP (qTAP) in *Arabidopsis* cell cultures in the presence and absence of *rac*-GR24. As baits, both SMXL7 and its modified version that is resistant to strigolactone-induced degradation were used. We show that the study of protein complexes involving a proteasome target as the bait protein is challenging, because of the bait degradation. Nevertheless, we demonstrate that the TAP technology combined with LFQ provides a sensitive platform with sufficient resolution to detect *rac*-GR24-dependent SMXL7 interactions in *Arabidopsis*.

## Materials and Methods

### Molecular Cloning

For all TAP constructs, cloning was performed by means of Gateway^®^ recombination (Thermo Fisher Scientific). The open reading frame (ORF) of SMXL7 was amplified from *Arabidopsis* cDNA with iProof^TM^ High-Fidelity DNA Polymerase (Bio-Rad) and Gateway^®^-specific primers. The PCR product flanked with attB sites was cloned in pDONR207 with the BP Clonase^®^ II enzyme mix (Invitrogen). The resulting entry vector was used to clone the bait into the destination vector pKNGS-rhino and pKCTAP for N- and C-terminal fusions, respectively, under the control of the 35S promoter (Van Leene et al., 2015) with the LR Clonase^®^ II Plus enzyme mix (Invitrogen). For the construction of the modified version of SMXL7 (hereafter designated ΔSMXL7), the Arg (R) at amino acid position 719 of the pDONR207-SMXL7 was mutated into a Thr (T), and the next five amino acids were deleted with the Spliced Overlap Extension PCR (SOE-PCR) (Higuchi et al., 1988). After sequence confirmation, the cloning steps were done in the same manner as for SMXL7. All primers used for cloning are listed in Supplementary Table 1.

### Cell Culture Transformation

The wild-type *Arabidopsis thaliana* (ecotype Landsberg *erecta*) cell suspension cultures were ordered at the Arabidopsis Biological Resource Center (ABRC). The cell cultures PSB-D (ABRC clone no. CCL84840) were transformed through cocultivation with *Agrobacterium tumefaciens* containing the N-terminal or both N- and C-terminal GSrhino fusions to SMXL7 and ΔSMXL7, respectively (Van Leene et al., 2007). After transformation, transgenic cell cultures were selected with a mixture of three antibiotics (25 μg/ml kanamycin, 500 μg/ml carbenicillin, and 500 μg/ml vancomycin) supplemented to the MSMO medium (4.43 g/L Murashige and Skoog basal salts with minimal organics [Sigma-Aldrich], 30 g/L sucrose, 0.5 mg/L α-naphtaleneacetic acid, 0.05 mg/L kinetin, pH 5.7). Three weeks after cocultivation, protein expression was analyzed. Cultures expressing the bait protein were subcultured in fresh MSMO medium at 21°C in a light/dark (16 h/8 h) regime with gentle agitation (130 rpm) and, subsequently, upscaled for TAP analysis.

### Western Blot Analysis

*Arabidopsis* cell cultures expressing *35S::GSrhino-SMXL7* and *35S::GSrhino-ΔSMXL7* were subcultured in 20 mL of fresh MSMO medium and grown at 25°C in the dark by gentle agitation (130 rpm). The synthetic strigolactone analog *rac*-GR24 was dissolved in acetone to a 10-mM concentration. Three days after subculturing, cell cultures were treated with 1 μM *rac*-GR24 or the equal volume of acetone (mock). Cell material was harvested before and at six time points after treatment (15 min, 30 min, 45 min, 1 h, 6 h, and 24 h). Total protein extract was prepared by adding the extraction buffer (see below for the buffer composition) to homogenized samples. Concentrations were determined by the Bradford assay (Bio-Rad). Of the total protein extract, 60 μg was separated by sodium dodecyl sulfate-polyacrylamide gel electrophoresis (SDS-PAGE) (12% Mini-PROTEAN^®^TGX^TM^ precast gels, Bio-Rad) and blotted on a polyvinylidene fluoride (PVDF) membrane (Trans-Blot^®^ Turbo^TM^ Mini PVDF Transfer, Bio-Rad) according to the manufacturer’s instructions. Blotted PVDF membranes were incubated in blocking buffer (3% [w/v] Difco^TM^ skimmed milk in TBS-T buffer [50 mM Tris-HCl, 150 mM NaCl, pH 8.0, 0.1% [v/v] Triton X-100]) for 1 h at room temperature on an orbital shaker. Afterward, the membranes were incubated with peroxidase-anti-peroxidase (PAP) antibody against the GS-rhino tag (Sigma-Aldrich) or anti-tubulin antibody (Sigma-Aldrich) to determine equal loading. The signal was captured by means of chemiluminescent substrates from the Western Lightning^®^ Plus Enhanced Chemiluminescence kit (Perkin-Elmer) and X-ray films (Amersham Hyperfilm ECL; GE Healthcare). The Precision Plus Protein^TM^ Dual Color Standards (Bio-Rad) was used as protein size marker.

### TAP Purification

Tandem affinity purification was carried out as described (Van Leene et al., 2015), with some modifications. Cell culture material was harvested after 10 min of treatment with 1 μM *rac*-GR24 or the equal volume of acetone. Total protein extract was prepared with extraction buffer (25 mM Tris-HCl, pH 7.6, 15 mM MgCl_2_, 150 mM NaCl, 15 mM *p*-nitrophenyl phosphate, 60 mM β-glycerophosphate, 0.1% [v/v] NP-40, 0.1 mM Na_3_VO_4_, 1 mM NaF, 1 mM phenylmethylsulfonyl fluoride [PMSF], 1 μM E64, EDTA-free Ultra complete tablet [1/10 mL; Roche Diagnostics], 0.1% [v/v] benzonase, and 5% [v/v] ethylene glycol). Total protein extract (25 mg) was incubated for 1 h at 4°C with 25 μL IgG-Sepharose 6 Fast Flow beads (GE Healthcare), pre-equilibrated in extraction buffer. After careful removal of the unbound fraction, the beads were washed on the Mobicol column with wash buffer (10 mM Tris-HCl, pH 7.6, 150 mM NaCl, 0.1% [v/v] NP-40, 0.5 mM EDTA, 1 μM E64, 1 mM PMSF, and 5% [v/v] ethylene glycol). The beads were incubated with 10 units of Rhinovirus 3C protease (GE Healthcare) for 1 h at 4°C. The IgG-eluted fraction was incubated with Streptavidin beads (GE Healthcare), equilibrated in wash buffer for 1 h at 4°C on a tube rotator. Bound complexes were eluted by streptavidin elution buffer (20 mM desthiobiotin in wash buffer) and proteins were concentrated by trichloroacetic acid (TCA) precipitation at 4°C overnight. In total, for each condition, four replicates were done for the cell cultures expressing *35S::GSrhino-SMXL7*, two for *35S::GSrhino-ΔSMXL7*, and two for *35S::ΔSMXL7-GSrhino.* The latter two were combined for the quantitative analysis.

### In-gel Protein Digestion

Purified protein samples were migrated on 4–12% gradient NuPAGE Bis-Tris gels (Life Technologies) for 7 min at 200 V and visualized with colloidal Coomassie Brilliant Blue G-250 (Sigma-Aldrich). The NuPAGE gel was de-stained twice for 1 h in high-performance liquid chromatography (HPLC)-grade water (Thermo Fisher Scientific) and incubated in 6.48 mM dithiothreitol and 50 mM NH_4_HCO_3_ in HPLC-grade water for 40 min to reduce disulfide bridges, and subsequently for 30 min in 54 mM iodoacetamide and 50 mM NH_4_HCO_3_ in HPLC-grade water in the dark for alkylation of the reduced thiol groups. After the gel had been washed for 30 min in 25 mL of HPLC-grade water, it was placed on a glass plate. The section containing all eluted proteins was cut out and sliced into 18 gel plugs. These plugs were dehydrated in 600 μL 95% (v/v) acetonitrile for 10 min, rehydrated with HPLC-grade water, and dehydrated again. Then, the gel plugs were rehydrated in trypsin digest buffer (1.125 mg trypsin [MS Gold; Promega], 50 mM NH_4_HCO_3_, 10% [v/v] acetonitrile in HPLC-grade water) for 30 min at 4°C. Subsequently, proteins were digested for 3.5 h at 37°C. The resulting peptide samples in the trypsin solution were sonicated for 5 min. The remaining gel plugs were dehydrated with 95% (v/v) acetonitrile for 10 min and added to the peptide solution. The overall resulting trypsin digest was vacuum-dried.

### LC–MS/MS Analysis

The obtained peptide mixture was analyzed by liquid chromatography-tandem MS (LC-MS/MS) with a tandem UltiMate 3000 RSLCnano system (Thermo Fisher Scientific) in-line connected to an LTQ Orbitrap Velos mass spectrometer (Thermo Fisher Scientific) through a Pneu-Nimbus dual-column source (Phoenix S&T). Peptides were first loaded on a trapping column (made in-house, 100 μm internal diameter [ID] × 20 mm length, 5 μm beads C18 Reprosil-HD [Dr. Maisch]) and then eluted and bound onto a reverse-phase analytical column (made in-house, 75 μm ID × 150 mm length, 5 μm beads C18 Reprosil-HD [Dr. Maisch]). The peptides were solubilized in 20 μL loading solvent (0.1% [v/v] trifluoroacetic acid in 98/2 water/acetonitrile [v/v]), of which 10 μL was loaded and separated with a linear gradient from 98% of solvent A (0.1% [v/v] formic acid in water) to 40% of solvent B (0.1% [v/v] formic acid in 20/80 [v/v] water/acetonitrile) in 30 min at a flow rate of 300 nL/min, and followed by a 5-min wash reaching 99% of solvent B. The mass spectrometer was operated in data-dependent, positive ionization mode, automatically switching between MS and MS/MS acquisition for the 10 most abundant peaks in a given MS spectrum. In the LTQ-Orbitrap Velos, full-scan MS spectra were acquired in the Orbitrap at a target value of 1E6 with a resolution of 60,000. The 10 most intense ions were then isolated for fragmentation in the linear ion trap, with a dynamic exclusion of 20 s. Peptides were fragmented after filling the ion trap at a target value of 1E4 ion counts. The background ion Asn3 at 445.120025 Da was used for internal calibration (lock mass).

### MS/MS Data Processing

All raw files were processed with the MaxQuant software (version 1.4.1.2) (Cox and Mann, 2008). The derived data were searched with the built-in Andromeda search engine against the *Arabidopsis thaliana* forward/reversed version of the TAIR10_pep_20101214 database containing also sequences of frequently observed contaminants, including human keratins, bovine serum proteins, or proteases. Carbamidomethylation of cysteines was selected as the fixed modification, whereas variable modifications were set to oxidation and acetylation (protein N-term). Trypsin∖P was selected as enzyme setting. Cleavage was allowed when arginine or lysine was followed by proline with two missed cleavages permitted. Matching between runs was enabled with a matching window time of 30 s. Relative, LFQ of proteins was selected by means of the MaxLFQ algorithm integrated into MaxQuant. With the minimum ratio count set to 1, the FastLFQ option was enabled, LFQ minimum number of neighbors was set to 3, and the LFQ average number of neighbors to 6, as per default. Proteins identified with at least one unique peptide were retained. The false discovery rate (FDR) for peptide and protein identifications was set to 1%, and the minimum peptide length was set to 7 amino acids. Detailed MaxQuant search parameters can be found in Supplementary Table 2. The mass spectrometry proteomics data have been deposited to the ProteomeXchange Consortium via the PRIDE (Vizcaino et al., 2016) partner repository with the dataset identifier PXD009083.

### Data Analysis

After MS data processing, LFQ values from the “proteinGroups.txt” output file of MaxQuant were further analyzed in the Perseus software (version 1.5.3.2). First, the reverse database hits, contaminants, and proteins identified only by modified peptides were filtered out. Then, log_2_ values were taken from the LFQ intensities, whereafter samples were grouped in ‘mock’ and ‘treatment.’ Proteins that did not contain at least four valid values in at least one group were filtered out and missing LFQ values were imputed/replaced by values from a normal distribution that were slightly lower than the lowest (log) value measured, as described (Smaczniak et al., 2012b; Wendrich et al., 2017). All the imputed missing values can be found in the Supplementary Data Sheet 1. For normalization on the bait level, the intensity values from the “proteinGroups.txt” of the MaxQuant output file were analyzed in the same manner as the LFQ values. Before the imputation step, the SMXL7 intensity was subtracted from the intensity value of each protein. A Student’s *t*-test was applied to determine statistical outliers between ‘mock’ and ‘treatment’ groups. The resulting differences between the means of the two groups [“log_2_(mock/treatment”)] and the negative log_10_ *P* values were plotted against each other in volcano plots. The multiple hypothesis testing problem was corrected with a permutation based FDR (0.05). The threshold value S0 was set at 0.1 by default.

### Yeast Two-Hybrid Analysis

Yeast two-hybrid (Y2H) analysis was done as described (Cuéllar et al., 2013) in two independent repeats. SMXL7 and ΔSMXL7 were cloned into the pB42AD Gateway vector (bait), whereas D14 and the N-terminal fragment of the TPL protein (TPL-N, Cuéllar Pérez et al., 2014) were cloned in the prey vector pGILDA. The polyethylene glycol (PEG)/lithium acetate method was used to co-transform the *Saccharomyces cerevisiae* EGY48 strain (Estojak et al., 1995) with the bait and prey. Transformants were selected on Synthetic Defined media containing galactose and raffinose (SD Gal/Raf) and lacking Ura, Trp, and His (Clontech). Three individual colonies were grown overnight in liquid cultures at 30°C and 10- and 100-fold dilutions were dropped on control media (SD Gal/Raf-Ura-Trp-His) and selective media containing X-Gal (Duchefa). To test the influence of the strigolactone analog on the interactions, 10 μM *rac*-GR24 or acetone (control) was added to the medium.

## Results

### *Arabidopsis* Cell Suspension Cultures Respond to *rac-*GR24 Treatment

**FIGURE 1:**
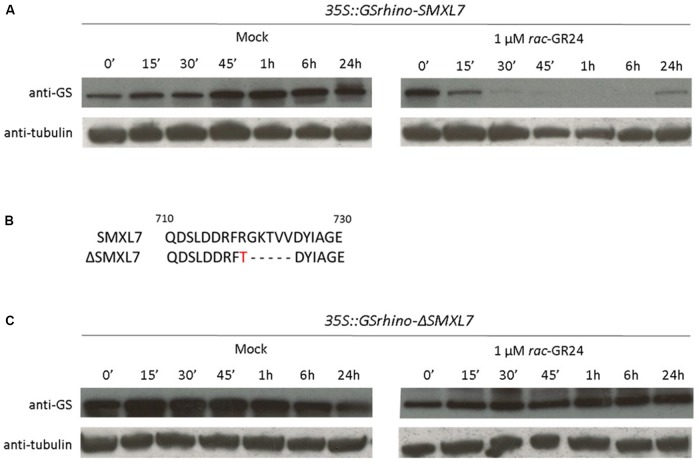
Response of *Arabidopsis* cell suspension cultures to *rac*-GR24. SMXL7 protein levels in cell cultures transformed with *35S::GSrhino-SMXL7* **(A)** or *35S::GSrhino-ΔSMXL7* **(C)** at different time points after treatment with either acetone (mock) or 1 μM *rac-*GR24 (‘minutes, h, hours after treatment). Detection was done with anti-GS (top) and anti-tubulin (bottom) antibodies, the latter as loading control. **(B)** Protein sequence alignment of the amino acid region of SMXL7 and ΔSMXL7. The ΔSMXL7 protein carries an Arg-to-Thr mutation followed by a deletion of residues 719-723 (Gly-Lys-Thr-Val-Val).

Cell cultures provide a good system to study PPIs involved in basic cellular pathways, because they offer a high protein yield and the possibility to perform hormone-induced studies (Van Leene et al., 2015). Indeed, they have already allowed the characterization of signaling complexes in different hormonal pathways, including, auxin, abscisic acid, and jasmonate (Pauwels et al., 2010; Fernández-Calvo et al., 2011; Irigoyen et al., 2014; Karampelias et al., 2016). However, this environment has never been used to study strigolactone signaling. To test whether the strigolactone pathway is active, the response of the cell cultures to *rac*-GR24 was tested. To this end, SMXL7 was N- and C-terminally fused with a GS^rhino^ tag and expressed in *Arabidopsis* cell cultures (see Materials and Methods). Only the N-terminal fusion (*35S::GSrhino-SMXL7*) yielded high protein levels, whereas no protein was detected for the C-terminal construct (Supplementary Figure 1). Therefore, only *35S::GSrhino-SMXL7* was utilized. The response to treatments with 1 μM *rac-*GR24 or with acetone (mock) was checked at different time points by Western blot analysis (**Figure 1**). The SMXL7 protein level decreased starting from 15 min after treatment and was partially recovered after 24 h (**Figure 1A**). Additionally, we tested a ΔSMXL7 allele that carries a mutation similar to that described in the *d53* allele in rice (**Figure 1B**) to render the protein resistant to *rac-*GR24-induced degradation (Jiang et al., 2013). Protein expression was detected in cell cultures for both N- and C-terminal fusions of GS^rhino^-tagged ΔSMXL7, although the levels were higher for the N-terminally tagged protein (Supplementary Figure 1). The ΔSMXL7 sensitivity to *rac*-GR24 was tested in cell cultures expressing *35S::GSrhino-ΔSMXL7* similarly as for *35S::GSrhino-SMXL7* and the *ΔSMXL7* protein level did not decrease upon treatment with *rac*-GR24, in agreement with previously published data (Jiang et al., 2013; Soundappan et al., 2015) (**Figure 1C**).

Taken together, these data demonstrate that *Arabidopsis* cell cultures respond to *rac-*GR24 and that all signaling components that are required for strigolactone-induced SMXL7 degradation are present in the cell cultures. Additionally, the change in the amino acid sequence of *ΔSMXL7* stabilized the protein after *rac*-GR24 treatment, confirming the importance of this region for protein degradation in both rice and *Arabidopsis* (Jiang et al., 2013; Soundappan et al., 2015).

### Quantitative TAP Reveals Changes in the SMXL7 Protein Complex Compositions

To examine the dynamics of the protein complexes formed around SMXL7 and their role in strigolactone signaling, we carried out TAPs in *Arabidopsis* cell cultures expressing GS^rhino^-tagged *SMXL7* or *ΔSMXL7* (see Materials and Methods). After the LC-MS/MS analysis of the TAP samples, spectra were searched with the MaxQuant software and resulted in the identification of 299 proteins for SMXL7 and 347 for ΔSMXL7. MaxLFQ was then used to quantify the identified proteins between the tested conditions over the four replicates. Further analysis was performed with the Perseus software as described (Smaczniak et al., 2012b). Changes in protein abundances were expressed after log_2_ transformation of protein LFQ intensity values. Proteins that were not assigned LFQ values in the MaxQuant search, because their abundance was below the detection limit under that specific condition or replicate, were assigned a value based on a normal distribution centered around the lowest detection limit of the measured intensities as described (Smaczniak et al., 2012b) (Supplementary Data Sheet 1). To evaluate the reproducibility of the analysis, scatter plots were made to calculate the correlations of LFQ values between replicates. Noteworthy, consistency between replicates is crucial for downstream statistical analysis. In our experiment, the Pearson correlation coefficients for all replicate pairs ranged from 0.765 to 0.959 for SMXL7 and from 0.891 to 0.976 for ΔSMXL7, indicating a good to very good reproducibility (Supplementary Figure 2).

Given the fast degradation of the SMXL7 protein (**Figure 1A**), TAP analysis was performed after 10 min of *rac-*GR24 treatment. To test the influence of this treatment, bait protein levels were compared between the conditions. The intensity of the SMXL7 protein was significantly lower in the hormone-treated samples than that in the mock samples (Supplementary Figure 3). In agreement with the Western blot analysis, the protein intensity levels of ΔSMXL7 were not influenced by the treatment with the strigolactone analog (Supplementary Figure 3).

**FIGURE 2:**
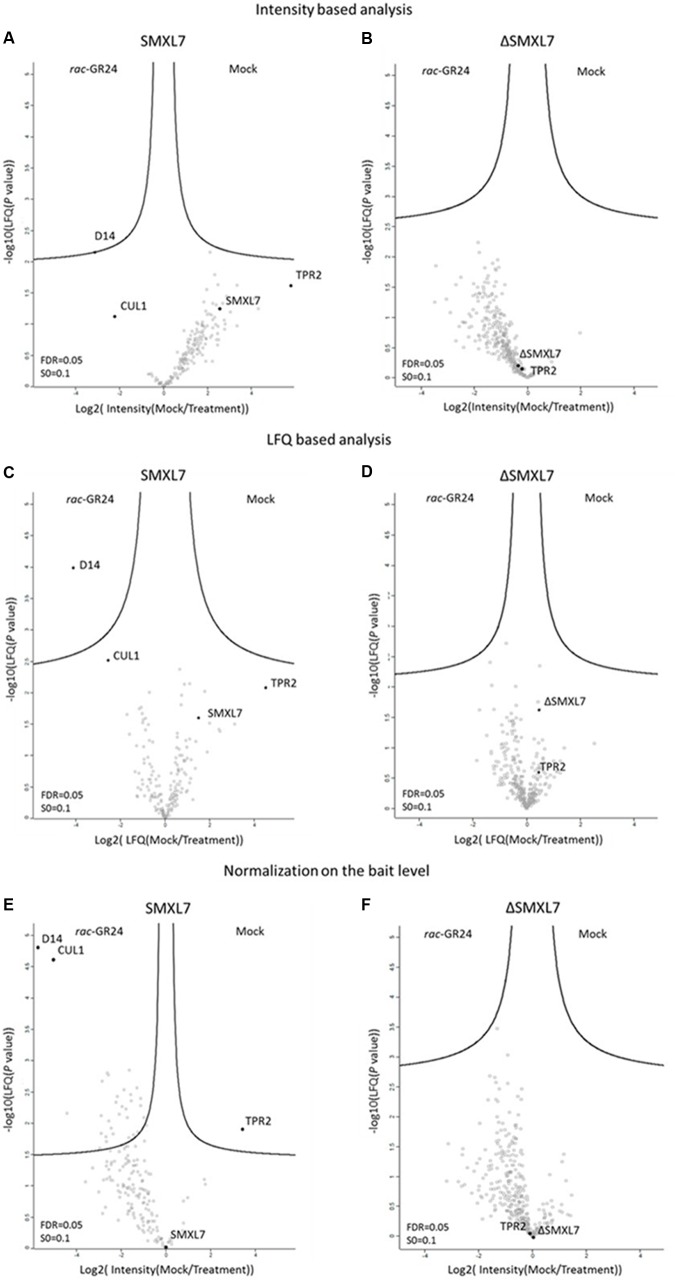
Dynamics of SMXL7 and ΔSMXL7 protein complexes. Volcano plots showing the distribution of all quantified proteins after filtering and statistical analysis, with their corresponding protein abundance ratios (Mock/Treatment) over the *t*-test *P*-value (FDR = 0.05, S0 = 0.1). Analysis was based on intensity values **(A,B,E,F)** or on LFQ values **(C,D)**. Protein distribution after normalization of the bait level **(E,F)**.

Statistical analysis was used to identify SMXL7-interacting proteins enriched in one of the tested conditions. In short, samples were assembled into either ‘mock’ or ‘treatment’ groups, with each group containing four biological repeats. In the first assessment, a *t*-test was done on the LFQ intensity values, allowing us to detect D14 as the only significant outlier. D14 was identified at significantly higher levels in *rac*-GR24-treated samples, indicating that the strigolactone receptor was recruited to the SMXL7 complex only in the presence of *rac*-GR24 (**Figure 2C**).

The LFQ values are normalized based on the overall protein abundance in the replicates. When non-normalized protein intensity values were used, the SMXL7 levels were clearly higher under mock conditions than those after treatment (**Figure 2A**), and this difference was also visible after LFQ application (**Figure 2C**). We then implemented another normalization step, in which we normalized on the bait level by subtracting the intensity values of the bait from those of the interacting proteins (**Figure 2E**). This drastically increased the number of preys identified as significantly associated with SMXL7 in the presence of *rac*-GR24; the total list of candidate interactors now contained 33 proteins (Supplementary Table 3). Many of the proteins associated with SMXL7 upon *rac*-GR24 treatment were related to the 26S proteasome (Supplementary Figure 4), hinting at a very active degradation process after hormone addition. Besides D14, CUL1 was also a protein that was significantly more associated with SMXL7 upon *rac*-GR24 treatment. The reason might be that in the current model of strigolactone signaling D14 recruits the SCF^MAX2^ complex of which CUL1 is one of its members (Stirnberg et al., 2007). Additionally, under the same condition, ubiquitin was significantly enriched, in line with the model of the ubiquitin-mediated degradation of SMXL7 upon strigolactone treatment. Interestingly, TPR2 was the only protein significantly more associated with SMXL7 under mock than in treatment conditions. It is thus likely that in response to *rac*-GR24, TPR2 might disassociate from the SMXL7 complex prior to the SMXL7 degradation. In the volcano plot based on the LFQ analysis, the distribution of both CUL1 and TPR2 is clearly separated from all the other quantified proteins, although not crossing the significance line (**Figure 2C**).

To assess whether the mutation of the amino acid region in ΔSMXL7 might influence the dynamics of protein complexes formed with SMXL7, we repeated the experiment with the ΔSMXL7 TAP constructs and applied the same statistical analysis. First, in the volcano plot based on non-normalized protein intensity values, the ΔSMXL7 levels were stable under both conditions (**Figure 2B**). Further, no proteins were detected as significantly more associated with the bait in one of the tested conditions (**Figures 2B,D,F**), demonstrating the importance of the mutated amino acid region for *rac*-GR24–induced interactions. D14 did not only no longer interact with ΔSMXL7 in a *rac*-GR24-dependent manner, but also it was not identified in any of the tested conditions (Supplementary Figure 5). Additionally, TPR2 was detected at the similar intensity level both in mock and after treatment with the strigolactone analog (Supplementary Figure 5).

**FIGURE 3:**
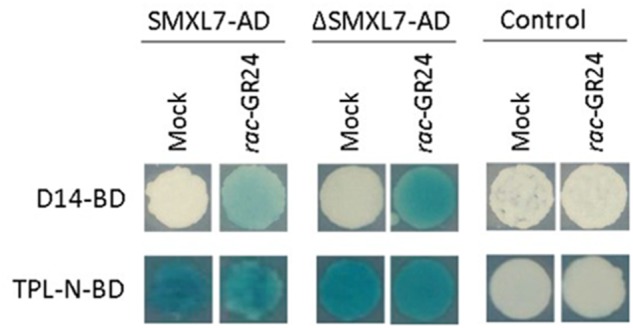
Interactions between SMXL7/ΔSMXL7 and D14 or TPL-N. The EGY48 (p8opLacZ) yeast (*Saccharomyces cerevisiae*) strain was cotransformed with D14 or TPL-N in pGILDA (BD) and SMXL7/ΔSMXL7 in pB42AD (AD) or pB42AD alone (control). Transformed yeasts were spotted on inducing medium containing Gal and Raf supplemented with 5-bromo-4-chloro-3-indolyl-β-D-galactopyranoside acid (X-Gal).

To validate the interaction of SMXL7 and ΔSMXL7 with D14 and TPR2, we used the Y2H LexA system, based on the detection of interactions through blue coloring of the yeast colony when spotted on selective SD-Ura-Trp-His medium supplemented with X-gal. In agreement with the qTAP analysis, SMXL7 interacted with D14 in a *rac*-GR24–dependent manner (**Figure 3**). The same was observed for ΔSMXL7 which is in contradiction with the results of the ΔSMXL7 qTAP analysis. To test the interaction with TPR2, we used the N-terminal fragment of TPL (TPL-N), consisting of the first 189 amino acids that are highly conserved between members of the TPL gene family, including TPL and TPR2. TPL-N contains the LisH, CTLH, and TOP domains that had previously been described as crucial for binding to the EAR motif and for mediating PPIs (Szemenyei et al., 2008; Nagels Durand et al., 2012; Cuéllar Pérez et al., 2014; Gonzalez et al., 2015). Y2H analysis confirmed the direct interaction of TPL-N with SMXL7 and ΔSMXL7 under both conditions. The SMXL7-TPL-N interaction was not disturbed after addition of *rac*-GR24, as would have been expected from the qTAP analysis. Taken together, although we can confirm the interaction between SMXL7, D14 and between SMXL7 and TPL, we cannot catch the entire *rac*-GR24-induced dynamics of the SMXL7-D14–TPR2 complex in the binary assay.

## Discussion

Here, we describe the use of LFQ MS-based analysis of samples generated by TAP to assess changes in PPIs in response to plant hormones. We used *Arabidopsis* cell cultures to set up the method, because this system had already been shown to be highly useful to study plant hormone signaling (Pauwels et al., 2010; Fernández-Calvo et al., 2011; Irigoyen et al., 2014; Karampelias et al., 2016).

Previously, the LFQ method has been proven to be efficient for the characterization of novel protein complexes in single-step AP-MS. Two independent protocols have been developed for plant research in which LFQ is used to distinguish between unspecific binding proteins and true interactors (Smaczniak et al., 2012b; Wendrich et al., 2017). Implementation of LFQ in AP-MS successfully allowed the identification of a critical regulator of the vascular development, the basic helix-loop-helix (bHLH) transcription factor dimer (De Rybel et al., 2013), the detection of the interaction network between five major floral homeotic MADS domain proteins (Smaczniak et al., 2012a), and the discovery of an association between PIN-FORMED (PIN) auxin efflux carriers and dynamin-related proteins (Mravec et al., 2011). Nevertheless, these protocols did not take into consideration the changes in the formed protein complexes upon perturbations, but show a stable view on all possible interactors.

In our approach, the basic idea is that TAP is performed on the cell cultures expressing a bait protein after they have been triggered with a particular plant hormone for a specific time. For data analysis, an LFQ algorithm, in this case MaxLFQ, was used in combination with statistical tests to identify the relevant interacting proteins. MaxLFQ requires sufficient numbers of stable background proteins to allow sample normalization (Pardo and Choudhary, 2012), thus many data points are needed to discriminate true interactors that associate differentially with a bait due to the treatment only. Although TAP provides relatively clean samples with rather low numbers of such background proteins, it has been already successfully used for the quantitative analysis of the changes in protein complexes formed around ANGUSTIFOLIA3 (ZmAN3) in maize (*Zea mays*); ZmAN3 was shown to engage in an interaction with distinct GROWTH-REGULATING FACTORs (GRFs) in the division zone when compared to the expansion zone of the growing leaf (Nelissen et al., 2015).

As a proof of concept, we focused on the protein complexes involved in strigolactone signaling. We demonstrated that *Arabidopsis* cell suspension cultures are suitable for studying the strigolactone pathway, because all the components required for the *rac*-GR24-dependent degradation of SMXL7 are present. Indeed, through Western blot and qTAP analysis, we detected a decrease in SMXL7 protein levels upon treatment with the strigolactone analog, indicative of *rac*-GR24-induced protein degradation, in agreement with former *in planta* studies (Soundappan et al., 2015; Wang et al., 2015; Liang et al., 2016). Additionally, consistent with previous reports, ΔSMXL7 that contains an amino acid change/deletion resembling that present in the *d53* allele in rice (Jiang et al., 2013) caused the protein to be resistant to *rac*-GR24-dependent degradation. As a result, the ΔSMXL7 protein levels under mock conditions and after hormonal treatment were the same.

SMXL7 as a direct target of SCF^MAX2^ is degraded upon *rac*-GR24 treatment in a D14-dependent manner (Soundappan et al., 2015; Wang et al., 2015). By means of the LFQ-based analysis, we identified an association of D14 with SMXL7 only in the presence of the hormone. Previously, the strigolactone-dependent interaction between SMXL7 (or D53 in rice) and D14 has been validated by different methods, including *in vitro* pull-down (Jiang et al., 2013), Förster resonance energy transfer (FRET) with fluorescence lifetime imaging microscopy (FLIM) (Liang et al., 2016), Y2H (Zhou et al., 2013; Wang et al., 2015), co-immunoprecipitation (Co-IP) (Wang et al., 2015), and bimolecular fluorescence complementation (BiFC) (Zhou et al., 2013). Here, we present the first MS-based view on this dynamic D14–SMXL7 interaction.

When proteins copurified with SMXL7 are compared in the presence and the absence of the strigolactone analog, a skewing of the protein intensity values occurs in the volcano plots toward an increased abundance under mock conditions. The reason might be that the *rac*-GR24-induced degradation of the bait causes a decrease in the intensity levels of all the proteins interacting with it and, consequently, they are less abundant after hormone treatment. As a result, the difference in bait protein levels between the tested conditions might hamper the detection of differentially interacting preys that follow the same trend as the bait protein levels. Therefore, we applied a normalization step on the intensity level of the bait protein itself rather than use a normalization based on background proteins (LFQ). Consequently, an increased number of proteins was identified that significantly associated with SMXL7 after treatment with the strigolactone analog. Although the list of candidate interactors might contain false positives, it might hint at processes that occur around the bait. Indeed, the STRING analysis revealed that some of these proteins are related to the 26S proteasome, indicating that the proteasomal degradation pathway is activated in response to *rac*-GR24. This observation is in agreement with the model in which the strigolactone action involves SCF^MAX2^-dependent ubiquitination of SMXL7 and its subsequent degradation by the 26S proteasome (Jiang et al., 2013; Zhou et al., 2013; Soundappan et al., 2015). The two most differentially accumulating proteins after normalization based on the bait levels, were indeed D14 and CUL1, implying the presence of the SCF^MAX2^ complex in close proximity of SMXL7 after addition of the strigolactone analog. Although these observations suggest that the normalization based on the bait level leads to a list of candidate interactors, from which at least a part is relevant and most likely bona fide interactors, further validation is required. When the same analysis was done with ΔSMXL7, no proteins belonging to the 26S proteasome-dependent protein turnover pathway were associated with the bait upon *rac-*GR24 treatment. Indeed, no proteins related to the 26S proteasome are expected to be recruited to the complex because ΔSMXL7 is resistant to *rac*-GR24-induced degradation, thereby blocking the strigolactone signaling (Soundappan et al., 2015; Wang et al., 2015).

In none of the tested conditions, MAX2 had been identified as an interactor of SMXL7. Independently of the *rac*-GR24 addition, an association between MAX2 and SMXL7 (or D53) had been reported by *in vitro* pull-down (Jiang et al., 2013) and Co-IP in *Arabidopsis* protoplasts (Wang et al., 2015), although a FRET-FLIM study indicated that the two proteins did not directly interact (Liang et al., 2016). The reason for the absence of MAX2 in our analysis might either be due to the low MAX2 expression level in the cell cultures, although it was high enough to induce strigolactone-dependent degradation of SMXL7, or to a too transient interaction between SMXL7 and MAX2 to survive the multi-step TAP protocol. Nevertheless, after normalization based on bait levels, CUL1 was significantly more associated with SMXL7 after treatment with *rac*-GR24. Although direct interaction with CUL1 is not expected because of its position in the SCF complex, it might hint at the presence of MAX2 near SMXL7 after the strigolactone analog addition.

Thus far, we gained insights into the composition of the SMXL7 protein complex that had previously often been shown by binary methods, confirming the power of the qTAP method. Interestingly, our analysis also revealed some results that do not fit with the current strigolactone signaling model. First, D14 was not found within the list of proteins copurified with ΔSMXL7 under any of the tested conditions, indicating the lack of interaction between these proteins in the cell cultures. This observation does not concur with our own Y2H data and with previous reports that used various binary PPI validation methods, such as Y2H, BiFC, pull-down, and FRET-FLIM (Jiang et al., 2013; Zhou et al., 2013; Liang et al., 2016). Second, according to the qTAP, the interaction between SMXL7 and TPR2 depends on *rac*-GR24, in contradiction with our own Y2H results and the mammalian two-hybrid assay used previously (Jiang et al., 2013). In the qTAP analysis of SMXL7, TPR2 was more associated with the bait under mock conditions than under the hormone treatment, particularly when normalization of the bait levels was applied. On the contrary, treatment with *rac*-GR24 had no influence on the interaction between ΔSMXL7 and TPR2, because the TPR2 level was similar under both conditions.

These discrepancies could be explained in different manners, of which one would be deviations on the stoichiometric balances between the proteins of the complex. Indeed, in most of the PPI methods, such as Y2H, BiFC, Co-IP, and FRET-FLIM, both tested proteins are overexpressed, whereas other potential complex components are absent (Y2H) or available at basal levels (BiFC, Co-IP, and FRET-FLIM). In this sense, qTAP is a unique approach, because it involves the overexpression of only one protein (bait) that retains stoichiometric relations with other members of the complex. This might be a possible reason for the inconsistency of the results obtained using qTAP compared to the other methods.

**FIGURE 4:**
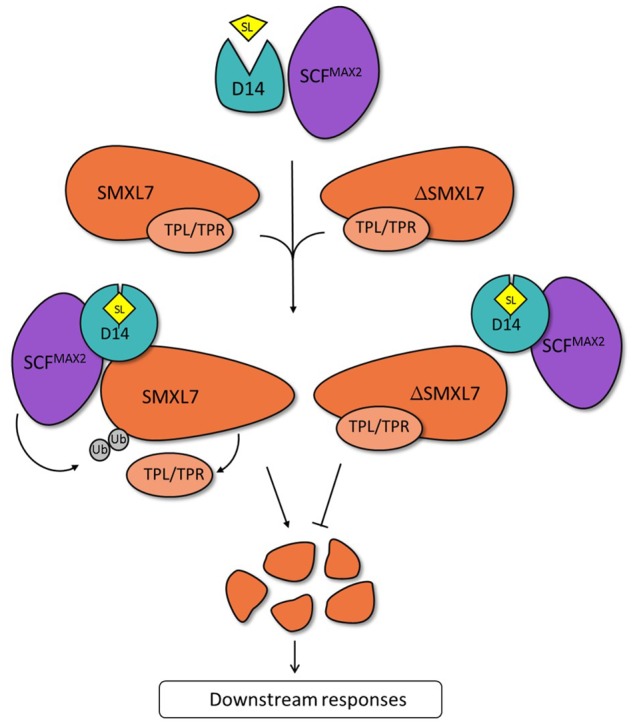
Proposed model for the strigolactone-induced dynamics of the SMXL7/ΔSMXL7 protein complex. In the presence of strigolactone, D14 and SCF^MAX2^ are recruited to SMXL7, whereas the TPL/TPR proteins dissociate from the complex. Subsequent ubiquitination of SMXL7 and its degradation by the 26S proteasome releases the repression of downstream responses. On the contrary, the ΔSMXL7-TPL/TPR interaction does not allow binding of the *rac*-GR24-bound D14 to ΔSMXL7, preventing the protein from degradation and activation of downstream responses.

Thus, the qTAP might data shed new light on the dynamics of protein complexes formed around SMXL7 in response to strigolactones. We hypothesize that after perception of *rac*-GR24, the TPL/TPR proteins might dissociate from the SMXL7 complex, potentially because an interaction with (an)other protein(s) interrupts or weakens the SMXL7-TPL/TPR association. Our analysis suggests that D14 could play this role, because its interaction profile is opposite that of TPR2. Thus, the conformational change of D14 triggered upon perception of strigolactones might enable binding to SMXL7 with such a high affinity that the TPL/TPR-SMXL7 interaction is disrupted. Subsequently, the ubiquitination and 26S proteasome-mediated degradation of SMXL7 would occur and downstream responses are activated (**Figure 4**) (Soundappan et al., 2015; Wang et al., 2015; Liang et al., 2016). Hence, the TPL/TPR-mediated repression is potentially released, not only because of the degradation of the repressors, but also because of the disruption of the interaction between SMXL7 and TPL/TPR proteins by the D14-to-SMXL7 binding. ΔSMXL7 might then act as a dominant-negative protein due to its stronger affinity to TPL/TPR proteins than the wild-type protein. As a result, the strigolactone-bound D14 cannot disrupt the ΔSMXL7-TPL/TPR interaction and, consequently, activate the downstream signaling (**Figure 4**). This hypothesis would explain the discrepancies observed between the qTAP data and the results obtained by binary methods, because such dynamic interactions can only be seen when more than two proteins are present in the assay. In the future, this hypothesis can be tested in various manners. Co-crystallization studies of SMXL7 together with TPL/TPR or D14 could indicate whether these proteins bind SMXL7 in the same domain, in which case sterical hindrance could dislocate TPL/TPR proteins upon strigolactone perception by D14. Additionally, binding studies with purified proteins in various combinations and conditions might shed light on their affinities.

## Conclusion

To conclude, given the progress in the MS field, mainly on increased sensitivities, combining TAP-MS with LFQ can become a powerful tool to study PPI dynamics. Tracking changes in the protein complex composition, as well as their assembly and disassembly during plant development can help to understand the role played by PPIs in several important plant growth processes. It would be interesting to use qTAP to resolve the complex dynamics in the signaling pathways of other plant hormones, because this approach has not been used yet. In the future, quantitative MS-based analysis of the interactions should be implemented in parallel with binary methods, because it provides novel insights into PPIs, accurately reflecting the cellular situation of their dynamic nature.

## Author Contributions

SS was the main author of the manuscript and performed all the experiments, except the molecular cloning that was done by ADK. The acquisition of the TAP data was done by GP. LB, AW, and GDJ were involved in MS/MS data analysis. F-DB kindly provided the synthetic strigolactone analog *rac*-GR24. KG and SG supervised the project and contributed to the writing of the manuscript.

## Conflict of Interest Statement

The authors declare that the research was conducted in the absence of any commercial or financial relationships that could be construed as a potential conflict of interest.
