# Supplementary material for: Quantitative Tandem Affinity Purification, an Effective Tool to Investigate Protein Complex Composition in Plant Hormone Signaling: Strigolactones in the Spotlight
Source: Front Plant Sci. 2018 Apr 26;9:528. doi: 10.3389/fpls.2018.00528 (PMC5932160; doi:10.3389/fpls.2018.00528)
Supplement: Supplementary file 1 [file Table_1.pdf]

Supplemental Table 1. Primers used in this study

Gene name	Primers	Use
SMXL7-Fw	GGGGACAAGTTTGTACAAAAAAGCAGGCTCAATGCCGACACCAGTAACCACG	Cloning
SMXL7-Rev1	GGGGACCACTTTGTACAAGAAAGCTGGGTATCAGATCACTTCGACTCTCG	Cloning
SMXL7-Rev2	GGGGACCACTTTGTACAAGAAAGCTGGGTAGATCACTTCGACTCTCG	Cloning
Δ*SMXL7-Fw*	CACAAGACAGTCTTGACGATAGATTCACAGATTACATTGCTGGCGAAGTGGCGAG	Mutagenesis
Δ*SMXL7-Rev*	CGCCACTTCGCCAGCAATGTAATCTGTGAATCTATCGTCAAGACTGTCTTGTGAC	Mutagenesis
D14-Fw	GGGGACAAGTTTGTACAAAAAAGCAGGCTCAATGAGTCAACACAACATCTTAG	Cloning
D14-Rev	GGGGACCACTTTGTACAAGAAAGCTGGGTATCACCGAGGAAGAGCTCGCCG	Cloning

Supplemental Table 2: MaxQuant search parameters

Parameter	Value
Version	1.4.1.2
Fixed modifications	Carbamidomethyl (C)
Decoy mode	revert
Special Aas	KR
Include contaminants	TRUE
MS/MS tol. (FTMS)	20 ppm
Top MS/MS peaks per 100 Da. (FTMS)	12
MS/MS deisotoping (FTMS)	TRUE
MS/MS tol. (ITMS)	0.5 Da
Top MS/MS peaks per 100 Da. (ITMS)	8
MS/MS deisotoping (ITMS)	FALSE
MS/MS tol. (TOF)	0.1 Da
Top MS/MS peaks per 100 Da. (TOF)	10
MS/MS deisotoping (TOF)	FALSE
MS/MS tol. (Unknown)	0.5 Da
Top MS/MS peaks per 100 Da. (Unknown)	10
MS/MS deisotoping (Unknown)	FALSE
PSM FDR	0.01
Protein FDR	0.01
Site FDR	0.01
Use Normalized Ratios For Occupancy	TRUE
Min. peptide Length	7
Min. score for unmodified peptides	0
Min. score for modified peptides	40
Min. delta score for unmodified peptides	0
Min. delta score for modified peptides	17
Min. unique peptides	1
Min. razor peptides	1
Min. peptides	1
Use only unmodified peptides and	TRUE
Modifications included in protein quantification	Acetyl (protein N-term); oxidation (M)
Peptides used for protein quantification	Unique
Discard unmodified counterpart peptides	TRUE
Min. ratio count	1
Site quantification	Use least modified peptide
Re-quantify	TRUE
Use delta score	FALSE
Ibaq	FALSE
iBAQ log fit	FALSE

MS/MS recalibration	FALSE
Match between runs	TRUE
Matching time window [min]	0.5
Alignment time window [min]	20
Find dependent peptides	FALSE
Fasta file	TAIR10_pep_20101214
Labeled amino acid filtering	TRUE
Site tables	Oxidation (M) sites.txt
Cut peaks	TRUE
Decoy mode	Revert
Special Aas	KR
Include contaminants	TRUE
RT shift	FALSE
Advanced ratios	FALSE
AIF correlation	0.47
First pass AIF correlation	0.8
AIF topx	20
AIF min mass	0
AIF SIL weight	4
AIF ISO weight	2
AIF iterative	TRUE
AIF threshold FDR	0.01

Supplemental Table 3. Proteins found significantly different between mock and treatment in SMXL7 TAP. *t* test analysis results from the Perseus software on a set of four independent TAP experiments on both mock and *rac*-GR24 treatment after intensity based normalization for bait.

**AGI code**	**Name**	***P value***
AT3G03990.1	α/β-Hydrolases superfamily protein	1.62E-05
AT4G02570.4	Cullin 1	2.55E-05
AT1G53750.1	Regulatory particle triple-A 1A	0.0014
AT5G35360.1	Acetyl Co-enzyme a carboxylase biotin carboxylase subunit	0.0016
AT5G16390.2	Chloroplastic acetylcoenzyme A carboxylase 1	0.0017
AT2G35635.1	Ubiquitin 7	0.0021
AT5G26860.1	Ion protease 1	0.0027
AT5G03850.1	Nucleic acid-binding, OB-fold-like protein	0.0029
AT5G23540.2	Mov34/MPN/PAD-1 family protein	0.0035
AT5G56030.1	Heat shock protein 81-2	0.0037
AT4G29040.1	Regulatory particle AAA-ATPase 2A	0.0040
AT5G20000.1	AAA-type ATPase family protein	0.0044
AT5G49910.1	Chloroplast heat shock protein 70-2	0.0046
AT3G05530.1	Regulatory particle triple-A ATPase 5A	0.0052
AT3G04120.1	Glyceraldehyde-3-phosphate dehydrogenase C subunit 1	0.0054
AT4G24280.1	Chloroplast heat shock protein 70-1	0.0059
AT5G43010.1	Regulatory particle triple-A ATPase 4A	0.0069
AT5G18230.1	Transcription regulator NOT2/NOT3/NOT5 family protein	0.0072
AT3G59540.1	Ribosomal L38e protein family	0.0075
AT5G02500.1	Heat shock cognate protein 70-1	0.0081
AT1G72370.2	40s Ribosomal protein SA	0.0081
AT1G71410.1	ARM repeat superfamily protein	0.0091
AT5G05620.1	γ-Tubulin complex protein 2	0.0120
AT3G16830.1	TOPLESS-related 2	0.0125
AT2G20580.1	26S Proteasome regulatory subunit S2 1A	0.0139
AT3G43300.2	HOPM interactor 7	0.0146
AT1G02080.2	Transcription regulators	0.0147
AT3G18190.1	TCP-1/cpn60 chaperonin family protein	0.0152
AT3G08530.1	Clathrin, heavy chain	0.0164
AT2G36160.1	Ribosomal protein S11 family protein	0.0172
AT3G09440.2	Heat shock protein 70 (Hsp 70) family protein	0.0182
AT1G71380.1	Cellulase 3	0.0195
AT3G53020.1	Ribosomal protein L24e family protein	0.0251
