# Supplementary material for: Quantitative Tandem Affinity Purification, an Effective Tool to Investigate Protein Complex Composition in Plant Hormone Signaling: Strigolactones in the Spotlight
Source: Front Plant Sci. 2018 Apr 26;9:528. doi: 10.3389/fpls.2018.00528 (PMC5932160; doi:10.3389/fpls.2018.00528)
Supplement: Supplementary file 3 [file Image_1.pdf]

Supplementary Material

Quantitative tandem affinity purification, an effective tool to investigate protein complex dynamics in plant hormone signaling: strigolactones in the spotlight

Sylwia Struk[1,2], Lukas Braem[1,2,3,4], Alan Walton[1,2,3,4], Annick De Keyser[1,2], François-Didier Boyer[5], Geert Persiau[1,2], Geert De Jaeger[1,2], Kris Gevaert[3,4] and Sofie Goormachtig[1,2*]

*** Correspondence:**
Sofie Goormachtig, Department of Plant Biotechnology and Bioinformatics, VIB-UGent, Center for Plant Systems Biology, Technologiepark 924, 9052 Gent, Belgium.
sofie.goormachtig@psb.vib-ugent.be

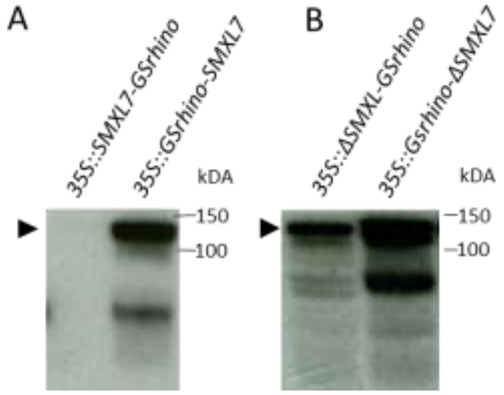

Supplemental Figure 1. Protein expression analysis of GS^{rhino}-tagged constructs. SMXL7 and ΔSMXL7 were N- and C-terminally tagged with the GS^{rhino} tag and overexpressed in *Arabidopsis* cell suspension cultures. Protein expression analysis was done before the up-scaling. Proteins were detected with the peroxidase anti-peroxidase (PAP) anti-GS tag antibodies during immunoblotting. The correct band is marked with an arrow.

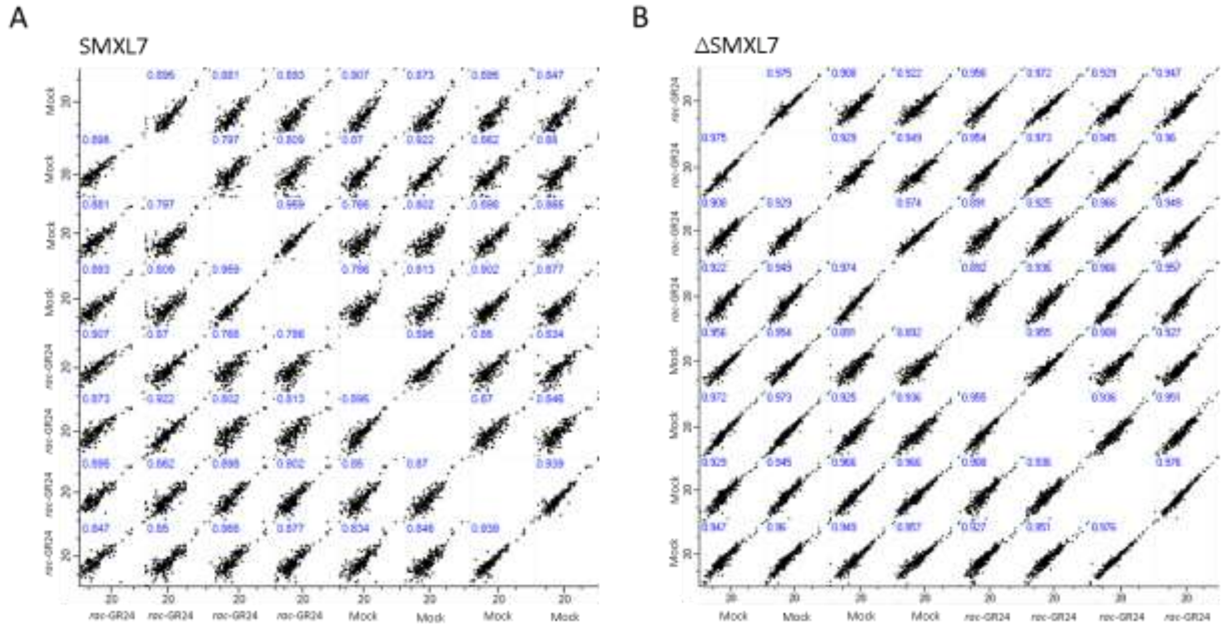

Supplemental Figure 2. Pearson correlation coefficients for *35S::SMXL7-GSrhino* and *35S::ΔSMXL7-GSrhino* combined with *35S::GSrhino-ΔSMXL7* samples mock or treated with *rac*-GR24. The matrix of 56 correlation plots reveals high correlations between LFQ intensities within replicates.

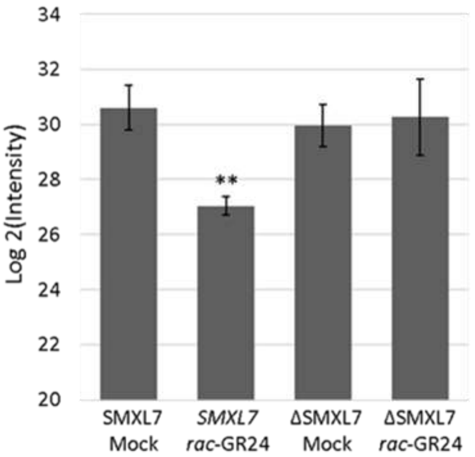

Supplemental Figure 3. Response of the bait SMXL7 and ΔSMXL7 proteins to the treatment with *rac*-GR24 in TAP samples. Average intensity values between all mock and treated replicates with standard deviation indicated. Asterisks indicate statistical significant differences ($P < 0.01$, *t* test).

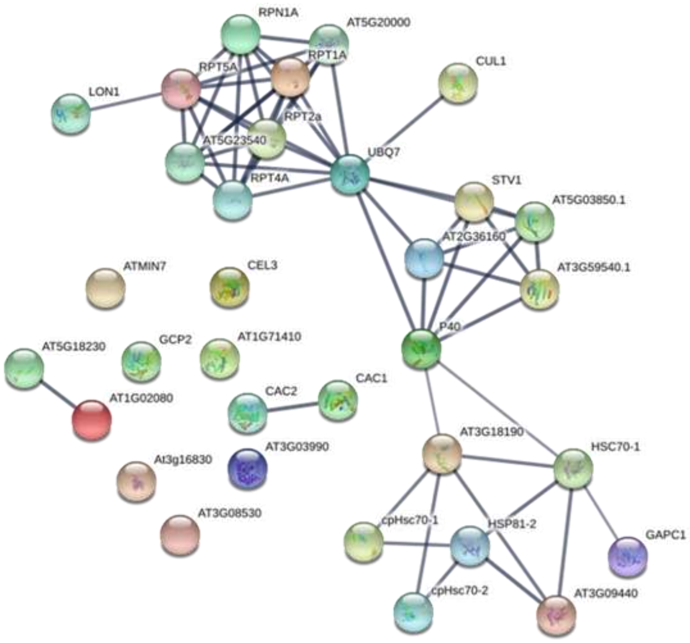

Supplemental Figure 4. STRING network revealing connections between the proteins associated with SMXL7. All the proteins that were significantly more associated with SMXL7 in *rac*-GR24-treated condition after normalization on the bait level were analyzed. Only high confidence interactions (score > 0.900) are shown. The three main complexes are involved in degradation mediated by 26S proteasome and 26S regulatory modules (top cluster), ribosome-related proteins (middle cluster), and chaperones involved in protein folding (bottom cluster).

	SMXL7 Mock				SMXL7 *rac*-GR24				ΔSMXL7 Mock				ΔSMXL7 *rac*-GR24			
replicate	1	2	3	4	1	2	3	4	1	2	3	4	1	2	3	4
CUL1	0.0	0.0	0.0	14.3	15.9	19.5	17.1	15.6	15.2	11.5	13.3	16.4	15.3	15.6	18.4	18.8
TPR2	21.2	23.4	18.9	20.0	0.0	20.0	0.0	0.0	18.2	19.0	20.6	19.0	19.4	17.7	20.5	20.6
D14	0.0	0.0	0.0	0.0	17.5	19.7	17.3	17.1	0.0	0.0	0.0	0.0	0.0	0.0	0.0	0.0

Supplemental Figure 5. Identification of CUL1, TPR2, and D14 in SMXL7 and ΔSMXL7 TAP experiments. The heat map displays the log2 intensity values of CUL1, TPR2 and D14 found in SMXL7 and ΔSMXL7 TAP experiments in mock and after *rac*-GR24 treatment in four repeats. Red 0.0 values indicate that the protein was not found in the repeat.
